# Salt stress responses and tolerance mechanisms in native desert plants of the UAE: growth, biochemical, and antioxidant perspectives

**DOI:** 10.3389/fpls.2026.1754346

**Published:** 2026-03-11

**Authors:** Sahara Abo Amin, Faisal Hayat, Mohammed Alyafei

**Affiliations:** Department of Integrative Agriculture, College of Agriculture and Veterinary Medicine, United Arab Emirates University, Al Ain, United Arab Emirates

**Keywords:** antioxidant enzymes, halophytes, *Lycium shawii*, photosynthetic efficiency, salinity, *Salvadora persica*

## Abstract

Soil salinization threatens productivity and ecosystem stability in arid regions, yet salinity-tolerance mechanisms of native UAE desert shrubs remain poorly resolved under field-like conditions. This study aimed to identify tolerance strategies and rank salinity resilience among *Lycium shawii*, *Salvadora persica*, *Calligonum comosum*, and *Haloxylon salicornicum*. Plants were grown in an outdoor pot trial under ambient desert conditions and irrigated with three salinity levels (ECw = 1.5, 10, and 25 dS m^-^¹). Severe salinity (25 dS m^-^¹) revealed clear interspecific divergence: *S. persica* and *L. shawii* maintained 100% survival, relatively stable water status and photosynthetic performance, and reduced membrane injury and lipid peroxidation. Their tolerance was associated with stronger osmotic adjustment (higher proline and soluble sugars), improved ion homeostasis (lower shoot Na^+^ accumulation and more stable K^+^ status relative to *H. salicornicum*), and enhanced antioxidant capacity (higher enzyme activities and radical scavenging). In contrast, *C. comosum* showed reduced survival (83.3%), marked dehydration, strong photosynthetic inhibition, weak antioxidant activation, and pronounced membrane damage, indicating limited high-salinity tolerance. *H. salicornicum* displayed intermediate performance, consistent with ion-handling–based tolerance but with higher physiological costs. Overall tolerance ranked *S. persica* > *L. shawii* > *H. salicornicum* > *C. comosum*. Correlation analysis and PCA supported coordinated contributions of water status, ion regulation, and oxidative protection to tolerance. These findings provide field-relevant mechanistic evidence to guide species selection for biosaline agriculture and saline-land rehabilitation in arid environments.

## 1 Introduction

Soil salinization is one of the most severe constraints to agricultural productivity, ecosystem stability, and sustainable development in arid and semi-arid regions. It threatens several United Nations Sustainable Development Goals (SDGs), particularly Zero Hunger (SDG 2), Clean Water and Sanitation (SDG 6), and Life on Land (SDG 15). Globally, more than one billion hectares of land are affected by salinity across more than 100 countries, and the extent of salt-affected soils continues to expand due to climate change, rising temperatures, seawater intrusion, and unsustainable irrigation practices. Beyond agriculture, soil salinization degrades biodiversity, alters ecosystem services, and limits the success of land rehabilitation and restoration programs (Singh, 2022). Salinity imposes two major stresses on plants: osmotic stress, which reduces water uptake and turgor, and ionic stress caused by excessive accumulation of Na^+^ and Cl^-^ in plant tissues. These stresses disrupt water relations, decrease stomatal conductance, impair CO_2_ fixation, and ultimately suppress photosynthetic efficiency and growth. Reduced carbon assimilation under salinity also enhances the generation of reactive oxygen species (ROS). Excess ROS can damage lipids, proteins, and DNA, leading to membrane instability, metabolic dysfunction, and cellular injury (Balasubramaniam et al., 2023).

To mitigate oxidative damage, plants activate complex defense systems that include enzymatic antioxidants such as superoxide dismutase (SOD), catalase (CAT), and peroxidases, as well as non-enzymatic antioxidants including carotenoids, phenolics, and other secondary metabolites. These systems work together with osmotic adjustment mechanisms (proline and soluble sugars accumulation) and ion regulation strategies (Na^+^ sequestration, K^+^ retention, and vacuolar compartmentalization) to maintain cellular homeostasis under salt stress (Abdulfatah, 2022; Mansoor et al., 2022; Kesawat et al., 2023; Sadiq, 2023; Bandodkar et al., 2025).

Plant species exhibit strikingly different responses to salinity. Glycophytic crops such as rice and wheat are highly salt-sensitive, whereas crops like barley and tomato show moderate tolerance. Tree crops, including olive and date palm, can tolerate salinity to some extent but still suffer yield reductions under high salt conditions (Sarkar and Sadhukhan, 2023; El Yamani and Cordovilla, 2024). In contrast, halophytes, including genera such as *Salicornia, Suaeda*, and *Atriplex* have evolved specialized adaptations that enable survival in highly saline environments. These include efficient Na^+^ sequestration in vacuoles, salt gland excretion, succulence, osmolyte accumulation, and enhanced antioxidant defenses. Consequently, halophytes are increasingly explored for biosaline agriculture, phytoremediation, and ecological restoration of degraded saline lands (Calone, 2022; Mehra et al., 2024; Soni et al., 2024). However, most mechanistic studies on salinity tolerance have focused on model species or a limited number of well-known halophytes, often under controlled greenhouse conditions. Moreover, greenhouse-based studies often overestimate salt tolerance because they exclude combined environmental constraints such as high radiation, temperature fluctuations, and evaporative demand, which strongly modify ion uptake, water relations, and oxidative stress responses in desert ecosystems. Therefore, tolerance rankings under controlled environments may not reflect plant performance under realistic arid-land conditions. As a result, there remains a critical knowledge gap regarding how native desert shrubs tolerate salinity under realistic environmental conditions.

In the United Arab Emirates (UAE), soil and groundwater salinity are intensifying due to seawater intrusion, over-extraction of groundwater, high evaporation rates, and the use of marginal-quality irrigation water. In several northern regions, groundwater salinity has been reported to reach up to 25 dS m^-^¹, posing serious risks to agriculture and natural ecosystems. Rising salinity has even affected traditionally salt-tolerant species such as date palms, with documented cases of dieback linked to salt accumulation (Elmahdy and Mohamed et al., 2023; Hammami et al., 2024). Field-based research in the UAE, including mangrove and halophyte trials irrigated with saline water, suggests that native shrubs can tolerate high ionic loads and may contribute to soil stabilization and salinity mitigation. Recent initiatives by the International Centre for Biosaline Agriculture (ICBA) and the Environment Agency- Abu Dhabi (EAD) have focused on screening native halophytes and implementing salinity-resilient revegetation programs across coastal and inland saline zones (Lyra et al., 2022; Choudhary et al., 2023; Friis and Killilea, 2023; Rhee et al., 2025). Within this context, four native UAE shrubs, *Salvadora persica, Lycium shawii, Calligonum comosum*, and *Haloxylon salicornicum* are of particular interest due to their ecological significance, traditional uses, and presumed salinity tolerance (**Table 1**). These species are well adapted to desert conditions and play important roles in habitat stabilization, biodiversity support, and potential biosaline agriculture (Brown and Feulner, 2023).

**Table 1:** Native UAE plant species used in the experiment, *Lycium shawii*, *Salvadora persica*, *Haloxylon salicornicum*, and *Calligonum comosum*, their habitats and ecological roles, key physiological traits supporting salinity adaptation, and known ethnobotanical and ecological uses.

Species	Habitat and ecological role	Physiological traits supporting salinity adaptation	Known uses	References
*L. shawii*	Rocky deserts and saline wadis	Strong osmotic adjustment, efficient antioxidant system, high membrane stability	Antidiabetic, anti-inflammatory, forage, saline land rehabilitation	Ali et al., 2020
*S. persica*	Coastal deserts and saline soils	High salt exclusion capacity, strong antioxidant defenses	Miswak (oral hygiene), antimicrobial, fodder, saline soil phytostabilization	Mekhemar et al., 2021
*C. comosum*	Sandy and gravel plains with moderate salinity	Moderate tolerance, weak antioxidant response, early growth decline	Forage, sand fixation, traditional medicine	Soliman et al., 2018
*H. salicornicum*	Highly saline arid zones and sabkha habitats	Moderate membrane stability, tolerates ionic stress	Fuelwood, rangeland rehabilitation, xerophytic landscaping	Panda et al., 2019

Despite growing interest in halophytes for biosaline agriculture and land rehabilitation, integrated mechanistic evidence under field-like desert conditions remains limited. Most existing studies either focus on model halophytes, single physiological traits, or greenhouse experiments, which do not fully capture plant responses under realistic environmental conditions (Todorović et al., 2022; Shahid and Alkandari, 2024). Consequently, there is insufficient knowledge to guide evidence-based species selection for large-scale saline land restoration in the UAE and similar arid regions. In particular, comparative multi-trait evaluations linking water status, ion regulation, and oxidative damage under field-like desert conditions remain largely unavailable for native Arabian shrubs. To address this gap, we tested the hypothesis that salt-tolerant native shrubs maintain higher water status and photosynthetic performance through coordinated ion regulation, osmotic adjustment, and antioxidant protection under realistic desert conditions. This study evaluates growth, water relations, ion regulation, photosynthetic traits, osmolytes, and oxidative stress responses of *L. shawii*, *S. persica*, *H. salicornicum*, and *C. comosum* under three irrigation salinity levels (ECw = 1.5, 10, and 25 dS m^-^¹) to identify species-specific tolerance strategies and guide saline-land rehabilitation in arid environments.

## 2 Materials and methods

### 2.1 Plant material and growth conditions

The experiment was conducted as an outdoor pot experiment under ambient desert conditions at Al Fo’ah Farm, United Arab Emirates University, in Al Ain, from October 2023 to May 2024. The site experiences a typical desert climate, with average daytime temperatures ranging from 28 to 35 °C and relative humidity between 30% and 50%. Uniform seedlings of four native desert species, including *L. shawii, S. persica, H. salicornicum, and C. comosum*, were obtained from Al Salamat Nursery, Al Ain. The soil at the site that used in the pots was classified as sandy (90% sand, 6% silt, 4% clay), with poor water-holding capacity and rapid drainage, typical of arid zones. The physicochemical properties of the experimental soil are summarized in **Table 2**. A completely randomized design (CRD) was implemented. Each species (15 plants per species) was assigned randomly to one of three irrigation salinity treatments: a control (EC_w_ = 1.5 dS m^-^¹), a moderate salinity level (EC_w_ = 10 dS m^-^¹), and a high salinity level (EC_w_ = 25 dS m^-^¹), with five biological replicates per treatment, where ECw refers to the electrical conductivity of the irrigation water. Salinity treatments were prepared by dissolving NaCl in the irrigation water as a standard salt source to impose controlled ionic stress and standardize treatments. Salinity was increased gradually over two weeks to minimize osmotic shock. Soil salinity was monitored weekly from the soil saturation extract (ECe) using a portable conductivity meter. Plants were grown individually in pots (50 cm height × 40 cm diameter) and arranged with sufficient spacing (≥1 m apart) to ensure uniform light penetration and to minimize edge effects. During the greenhouse stage (Acclimatation stage; August to September 2023), plants were grown under natural sunlight supplemented with LED lights (200 µmol/m²/s) to maintain a photoperiod of 14 h light/10 h dark at 28 ± 2 °C. In the field stage (Salinity stress stage; October 2023 to May 2024), light intensity ranged from 1200 to 2000 µmol/m²/s during daylight hours, with an average natural photoperiod of 11 to 13 hours across the winter–spring season.

**Table 2:** Physicochemical properties of the experimental soil (0–30 cm depth) at the experiment site in Al Ain, UAE, including texture, pH, electrical conductivity, organic matter, and macro-nutrient contents, with methods and references used for each parameter.

Parameter	Value	Method	Reference
Soil texture	Sandy (90% sand, 6% silt, 4% clay)	Hydrometer method	Huluka and Miller, 2014
Water-holding capacity	Low	Visual/drainage observation	Mulidzi et al., 2016
pH	8.0	pH meter	Al-Busaidi et al., 2005
Electrical conductivity (ECe)	1.1 dS m^-^¹	EC meter	Miller and Curtin, 2006
Organic matter content	<0.5%	Walkley-Black method	El Mouridi et al., 2023
Nitrogen (mg kg^-^¹)	28	Kjeldahl digestion	Bremner, 1960)
Phosphorus (mg kg^-^¹)	7.4	Olsen method	Sims, 2000
Potassium (mg kg^-^¹)	93	Flame photometry	Ullah et al., 2022

### 2.2 Observations of morphological traits

Morphological and growth parameters were recorded under control and salinity stress treatments, including plant height, leaf area (or segment length for leafless species), root to shoot ratio and survival rate. Plant height was measured using a meter scale. For species with leaves, leaf area was determined from five representative plants per treatment using the manual tracing method on graph paper. For species without leaves, the mean stem segment length was recorded. Fresh weight was measured immediately after harvest, and samples were then wrapped in aluminum foil and oven-dried at 80 °C for 48 hours to determine dry weight. Plants were separated into shoots and roots before weighing, and root-to-shoot ratio was calculated as root dry weight divided by shoot dry weight. Survival rate was calculated as the percentage of healthy plants remaining in each treatment at the end of the experiment. In addition, a salt injury index was scored visually on a 1–5 scale (1 = healthy plants with no necrosis; 2 = 1–25% necrotic leaf area; 3 = 26–50%; 4 = 51–75%; 5 = 76–100% necrotic area or plant death), following Zhen et al (Zhen et al., 2010).

### 2.3 Photosynthetic pigments and gas exchange parameters

Total chlorophyll, chlorophyll a, chlorophyll b, and carotenoid contents were estimated from fully expanded leaves using 85% acetone extraction following Palta (Palta, 1990). Absorbance was recorded at 663, 645, and 440 nm using a spectrophotometer (Helios Alpha, Thermo Scientific), and pigment concentrations were calculated using the MacKinney equations (Marker, 1972), and values were expressed on a fresh weight basis (µg g^-^¹ FW).

Gas exchange parameters, including net photosynthetic rate (A), stomatal conductance (gs), transpiration rate (E), and vapor pressure deficit (VPD_leaf_), were measured using a portable infrared gas analyzer (LI-6400XT Portable Photosynthesis System., USA) on the first fully expanded leaf of each plant. Measurements were taken under ambient light and temperature conditions, with three biological replicates per treatment.

### 2.4 Relative water content, membrane stability, and lipid peroxidation

Leaf relative water content (RWC) was determined following Mullan and Pietragalla (Mullan and Pietragalla, 2012) using five leaves per treatment. Fresh weight (FW), turgid weight (TW), and dry weight (DW) were measured, and RWC was calculated using Equation 1.

(1) $$RWC\;\left(\%\right)=\frac{\left(FW-DW\right)}{\left(TW-DW\right)}\times 100$$

Membrane stability was assessed by measuring electrolyte leakage (EL) following Dionisio-Sese and Tobita (Dionisio-Sese and Tobita, 1998). Fresh leaves (0.2 g) were incubated in deionized water for 2 h at 32 °C to record initial conductivity (EC1), then autoclaved and cooled to measure final conductivity (EC2). EL (%) was calculated using Equation 2.

(2) $$EL\;\left(\%\right)=\frac{EC1}{EC2}\times 100$$

Lipid peroxidation was measured by estimating malondialdehyde (MDA) content following the thiobarbituric acid (TBA) method (Schmedes and Hølmer, 1989). Absorbance was recorded at 532 and 600 nm, and MDA was calculated using an extinction coefficient of 155 mM^-^¹ cm^-^¹ and expressed as nmol/g fresh weight.

### 2.5 Biochemical parameters and antioxidant activities

Antioxidant enzyme activities, including catalase (CAT), peroxidase (POD), polyphenol oxidase (PPO), and superoxide dismutase (SOD), were analyzed from leaf extracts using standard spectrophotometric methods described by Beers and Sizer (Beers and Sizer, 1952), Chance and Maehly (Chance and Maehly, 1955), Mayer et al (Mayer et al., 1966), and Beauchamp and Fridovich (Beauchamp and Fridovich, 1971), respectively. Triplicate samples were homogenized in 0.1 M sodium phosphate buffer (pH 7.0) and centrifuged at 12,000×g for 15 minutes at 4 °C and absorbance was measured at appropriate wavelengths for each assay. Enzyme activities were expressed on a protein basis (U/mg protein). DPPH (radical scavenging activity) was determined using the method of Blois (Blois, 1958) with minor modifications. Absorbance was recorded at 517 nm, and the percentage of radical scavenging activity was calculated relative to a methanol blank. Total soluble proteins were estimated using the Bradford method (Bradford, 1976), with bovine serum albumin as the standard.

### 2.6 Ionic content and soluble sugars

Ionic concentrations were determined in leaf, stem, and root tissues using Inductively Coupled Plasma Mass Spectrometry (ICP-MS). Oven-dried samples (0.5 g) were digested with a 9:4 mixture of nitric and perchloric acids using a microwave digestion system (CEM-MARS 6), diluted to 50 mL with deionized water, and filtered. Macronutrients (K, P) and micronutrients (Fe, Mn, Zn, Na) were quantified using standard calibration curves and expressed as mg kg^-^¹ DW. Total soluble sugars were estimated following ethanol extraction and reaction with Anthrone reagent. Absorbance was measured at 620 nm, and sugar concentration was calculated against a glucose standard curve and expressed as mg/g dry weight.

### 2.7 ROS, H_2_O_2_, proline, and phenolic compounds

Hydrogen peroxide (H_2_O_2_) content was estimated following Velikova et al (Velikova et al., 2000). by reacting leaf extracts with KI and measuring absorbance at 390 nm. Reactive oxygen species (ROS) were quantified using the nitro blue tetrazolium (NBT) method as described by Jambunathan (Jambunathan, 2010), with absorbance recorded at 530 nm. ROS values were expressed as arbitrary absorbance units per g fresh weight (AU/g FW).

Proline concentration was determined using the acid ninhydrin method of Bates et al (Bates et al., 1973), and absorbance was measured at 520 nm after toluene extraction. Total phenolic content was quantified using the Folin–Ciocalteu method from methanol-extracted leaf samples, with absorbance recorded at 765 nm after incubation at 40 °C for 30 minutes. Total phenolic content was expressed as gallic acid equivalents (mg GAE g^-^¹ DW) using a gallic acid calibration curve.

### 2.8 Statistical analysis

Data were tested for normality and homogeneity of variance prior to analysis. Differences among all treatment combinations (species × salinity) were evaluated using one-way ANOVA, considering each combination as an independent group. When significant differences were detected, means were separated using Tukey’s HSD test at P ≤ 0.05. Statistical analyses were performed using R Studio (version 2022.07.1; R Core Team, Vienna, Austria), and graphical representations were prepared with SigmaPlot (version 12.0) and R Studio. Morphological and water-relation traits were measured using five biological replicates per treatment (n = 5). Gas-exchange parameters were measured on three biological replicates per treatment (n = 3) due to instrument measurement time limitations. For biochemical analyses, tissues from independent plants were analyzed separately (n = 6 biological replicates). All assays were performed with three technical replicates per biological replicate. Data are presented as means ± standard deviation (SD), unless otherwise stated. For mineral nutrient concentrations (**Table 3**), each organ was analyzed separately due to inherent physiological differences among plant organs. Therefore, nutrient concentrations were compared across the combined groups (species × salinity) using one-way ANOVA followed by Tukey’s HSD test (P ≤ 0.05).

**Table 3:** Selected macronutrient (K, P) and ion/micronutrient (Na, Fe, Zn, Mn) concentrations (mg kg^-^¹ DW) in roots and shoots of *Lycium shawii*, *Salvadora persica*, *Haloxylon salicornicum*, and *Calligonum comosum* under control, medium, and severe salinity conditions.

Species × treatment × organ	Na	K	P	Fe	Zn	Mn
*L. shawii* control roots	2987.3 l	4655.6 j	1313.5 l	1841.1 a	64.5 g	64.9 d
*L. shawii* control shoots	5307.2 k	5461.2 h	1480.8 j	638.5 c	108.3 c	28.5 g
*L. shawii* medium roots	7987.7 j	5186.3 i	1584.1 k	705.6 g	93.9 b	38.4 h
*L. shawii* medium shoots	7225.7 j	3225.9 k	1400.6 k	1379.7 a	93.1 e	77.4 b
*L. shawii* severe roots	6119.6 k	3437.6 l	2007.1 i	926.7 e	98.8 a	36.1 j
*L. shawii* severe shoots	7752.8 i	3371 j	2292.6 e	739.7 b	155.2 a	33.9 f
*S. persica* control roots	12000.1 i	10581.8 d	2157.2 h	440.1 j	90.7 c	24.7 k
*S. persica* control shoots	14835.6 f	6133.3 g	2383.9 d	97.8 j	81.6 f	23.9 j
*S. persica* medium roots	21284.4 c	12765.5 b	2396.7 g	212.2 l	62.5 i	23.1 l
*S. persica* medium shoots	22883.5 d	15893.2 a	3066 b	93.3 k	45.1 k	17.1 k
*S. persica* severe roots	19919.3 d	7195.4 e	1819.1 j	1000.2 d	58.5 j	44 g
*S. persica* severe shoots	14174 g	3822.6 i	1654.4 i	167.4 i	50.1 j	24.6 i
*H. salicornicum* control roots	31479.3 a	17553.3 a	3416.7 b	1561.7 b	89.2 d	158.2 a
*H. salicornicum* control shoots	26946.5 c	13115.9 b	2524.3 c	280.5 e	125.5 b	68.7 d
*H. salicornicum* medium roots	24225.7 b	12121.9 c	3137.1 d	1134.2 c	62.85 h	132.7 b
*H. salicornicum* medium shoots	32139.9 b	12551.4 c	2099.9 g	234.5 g	101.4 d	76.7 c
*H. salicornicum* severe roots	16972 f	6690.5 f	2857.5 e	706.7 f	36.5 l	107.2 c
*H. salicornicum* severe shoots	37333.2 a	11986.8 d	1675.5 h	188.5 h	77.2 i	84.7 a
*C. comosum* control roots	12582 h	5328.5 h	2598.5 f	500.8 h	75.5 f	54.3 e
*C. comosum* control shoots	7759.1 i	3361 j	2297.4 e	738.7 b	154.2 a	33.7 f
*C. comosum* medium roots	18413.4 e	6024.3 g	4562.5 a	391.8 k	84.9 e	38.1 i
*C. comosum* medium shoots	13960.2 h	8343.9 e	3278.3 a	327.8 d	77.9 h	28.2 h
*C. comosum* severe roots	15467.4 g	4548.2 k	3156.4 c	467.5 i	56.4 k	45.6 f
*C. comosum* severe shoots	17450.2 e	7551.5 f	2240.3 f	245.1 f	78 g	41 h

Data were analysed using one-way ANOVA with the combined factor (species × salinity treatment × organ) for each nutrient. Different lowercase letters indicate statistically significant differences among all group means across the entire table for that nutrient according to Tukey’s HSD test (P ≤ 0.05).

## 3 Results

### 3.1 Survival rate

Survival differed significantly among species (P< 0.05) at the highest salinity level (25 dS m^-^¹). Under control conditions, all species exhibited 100% survival. Under severe salinity, *L. shawii*, *S. persica*, and *H. salicornicum* maintained full survival (100%), whereas *C. comosum* showed significantly lower survival under severe salinity (83.3%).

### 3.2 Plant height

**Figure 1:**
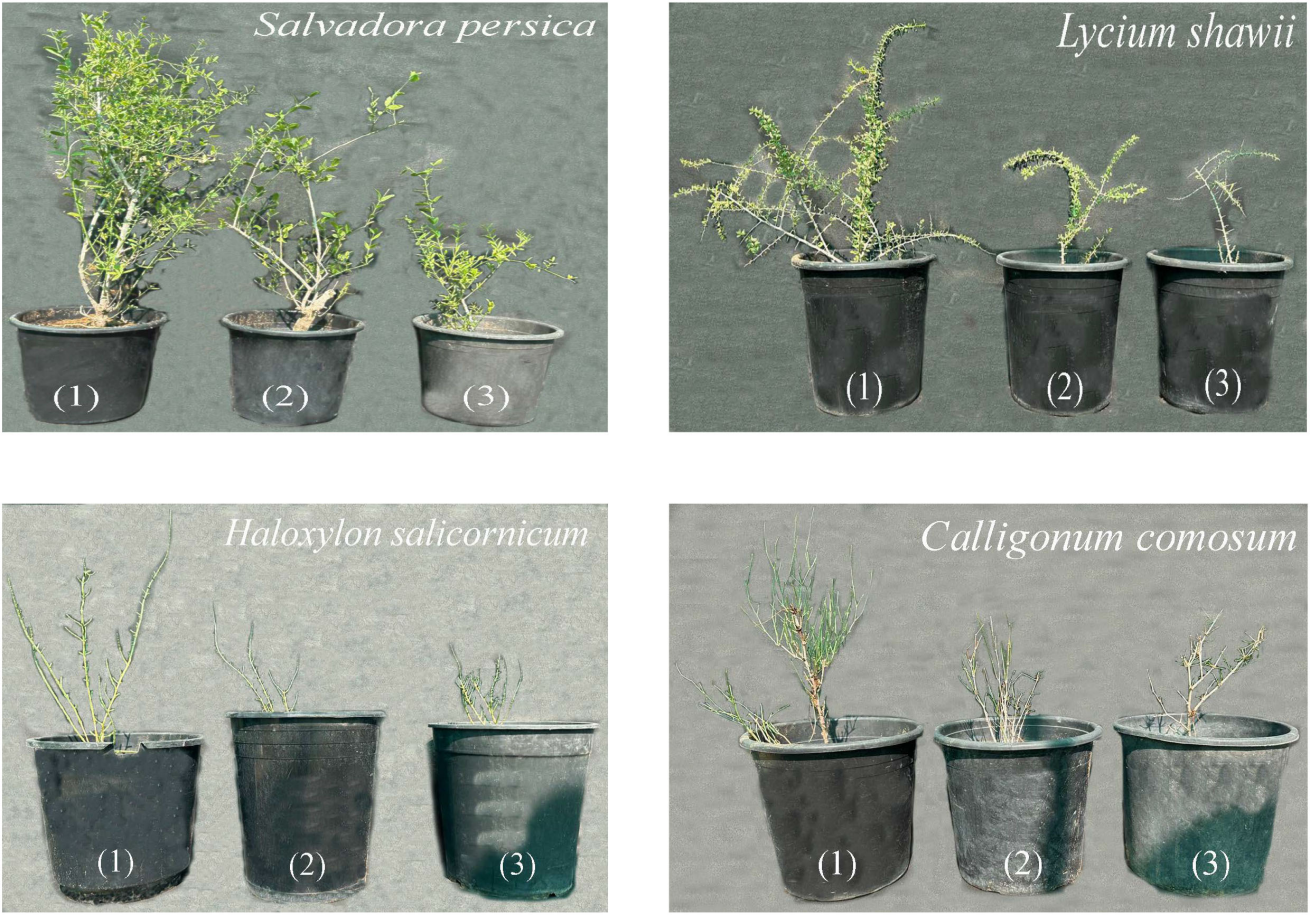
Representative photographs of four native UAE shrubs (*Calligonum comosum*, *Haloxylon salicornicum*, *Lycium shawii*, *and Salvadora persica*) under control, 10 and 25 dS m^-^¹ salinity treatments. Images document visual phenotypic responses only; quantitative differences are presented in subsequent figures.

**Figure 2:**
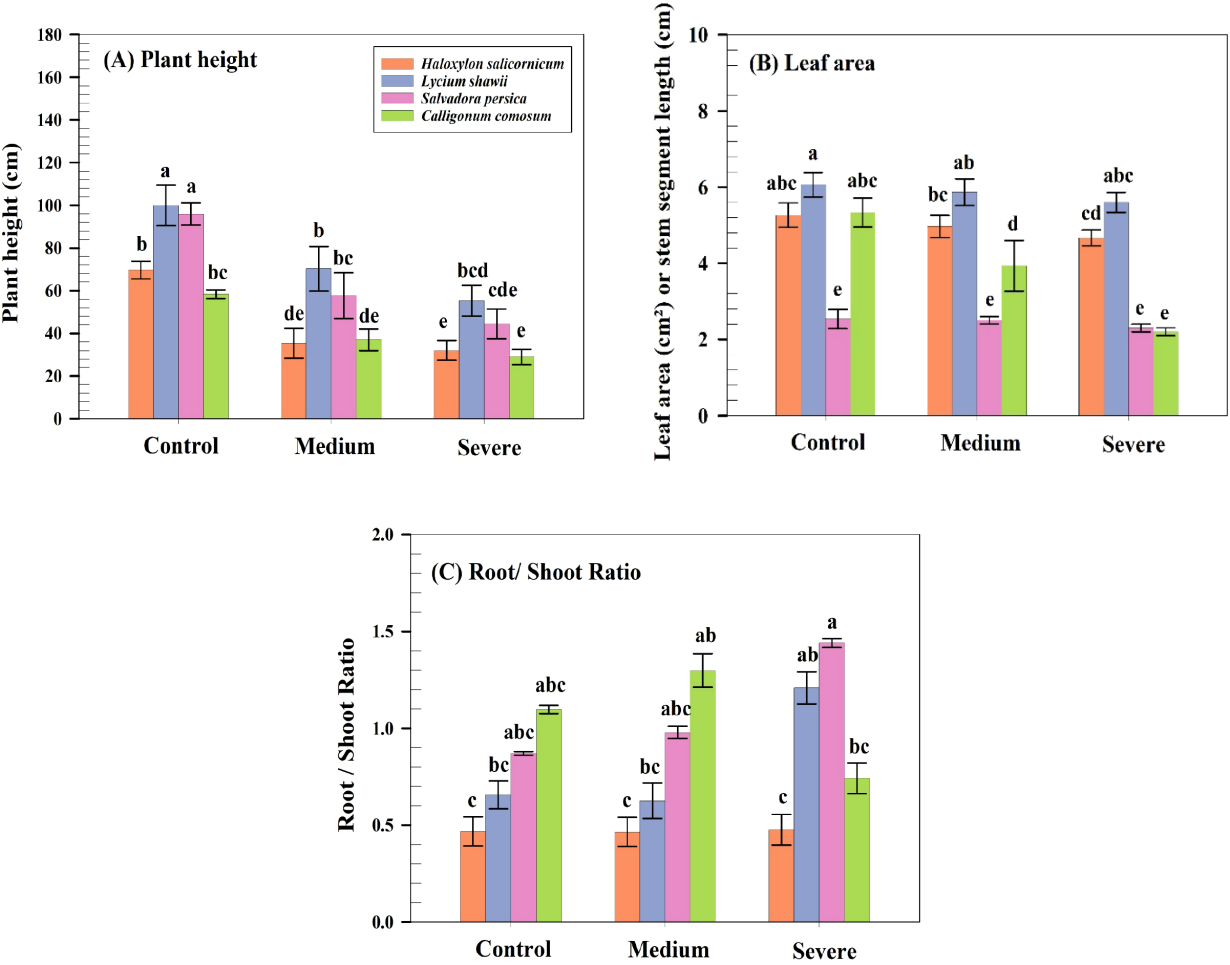
Effects of salinity on morphological traits of the studied species. **(A)** Plant height (cm). **(B)** Leaf area in *S. persica* and *L. shawii*, and stem segment length in *C. comosum* and *H. salicornicum* (cm² or cm). **(C)** Root-to-shoot ratio, reflecting biomass allocation under salinity. Data are means ± SD (n = 5).

Plant height differed significantly among species (P< 0.05), with the greatest reduction observed in *C. comosum* under severe salinity (from 58.3 to 29.0 cm), followed by *H. salicornicum* (69.7 to 32.0 cm), *S. persica* (96.0 to 44.3 cm), and *L. shawii* (100.0 to 55.3 cm; **Figures 1**, **2**). The salt-injury index increased progressively with salinity in all species. Under severe salinity, *C. comosum* exhibited the highest salt injury index, *H. salicornicum* showed intermediate salt injury index, and *S. persica* and *L. shawii* displayed the lowest salt injury index levels (**Table 4**).

**Table 4:** Survival (%) and salt-injury index (1–5) of *Lycium shawii*, *Salvadora persica*, *Haloxylon salicornicum*, and *Calligonum comosum* under control (1.5 dS m^-^¹), medium (10 dS m^-^¹), and severe (25 dS m^-^¹) salinity treatments (n = 5).

Species	Survival control (%)	Survival 10 dS m^-^¹ (%)	Survival 25 dS m^-^¹ (%)	Salt-Injury index control	Salt-Injury index 10 dS m^-^¹	Salt-Injury index 25 dS m^-^¹
*L. shawii*	100	100	100	1	1	1.5
*S. persica*	100	100	100	1	1	1.2
*H. salicornicum*	100	100	100	1	2	3
*C. comosum*	100	96.7	83.3	1	2.5	4

The salt-injury index reflects visible necrosis and plant death.

### 3.3 Leaf area and stem segment length

Leaf area or stem segment length varied significantly among species (p ≤ 0.05). In leafy species, *S. persica* showed a slight but significant reduction (2.53 to 2.30 cm²), whereas *L. shawii* exhibited a moderate decline (6.07 to 5.60 cm²). Among leafless species, *C. comosum* displayed a marked reduction in stem segment length (5.33 to 2.20 cm), whereas *H. salicornicum* showed a smaller decrease (5.27 to 4.67 cm). Tukey’s *post-hoc* comparisons confirmed significant treatment effects within each species (**Figure 2B**).

### 3.4 Biomass production

Biomass accumulation differed significantly among species (P< 0.05), with species-specific responses. *L. shawii* showed moderate reductions in shoot fresh weight (22.5 to 12.4 g) and a slight decrease in root dry weight (5.6 to 5.1 g) under severe salinity. In contrast, *C. comosum* experienced the greatest biomass loss, with root fresh weight declining from 30.7 to 3.9 g and root dry weight from 26.1 to 2.4 g. *S. persica* maintained relatively stable shoot and root biomass across treatments (P > 0.05; **Figure 2C**).

### 3.5 Root-to-shoot ratios

Root-to-shoot ratios varied significantly among species (P< 0.05), with contrasting patterns among species. Under severe salinity, *L. shawii* exhibited the highest root-to-shoot ratio. Conversely, *C. comosum* showed a significant decline in root-to-shoot ratio (from 1.10 to 0.74), resulting in the lowest value among all species under severe salinity (**Figure 2C**). These differences were confirmed by Tukey’s HSD comparisons.

### 3.6 Effects of salinity on relative water content

**Figure 3:**
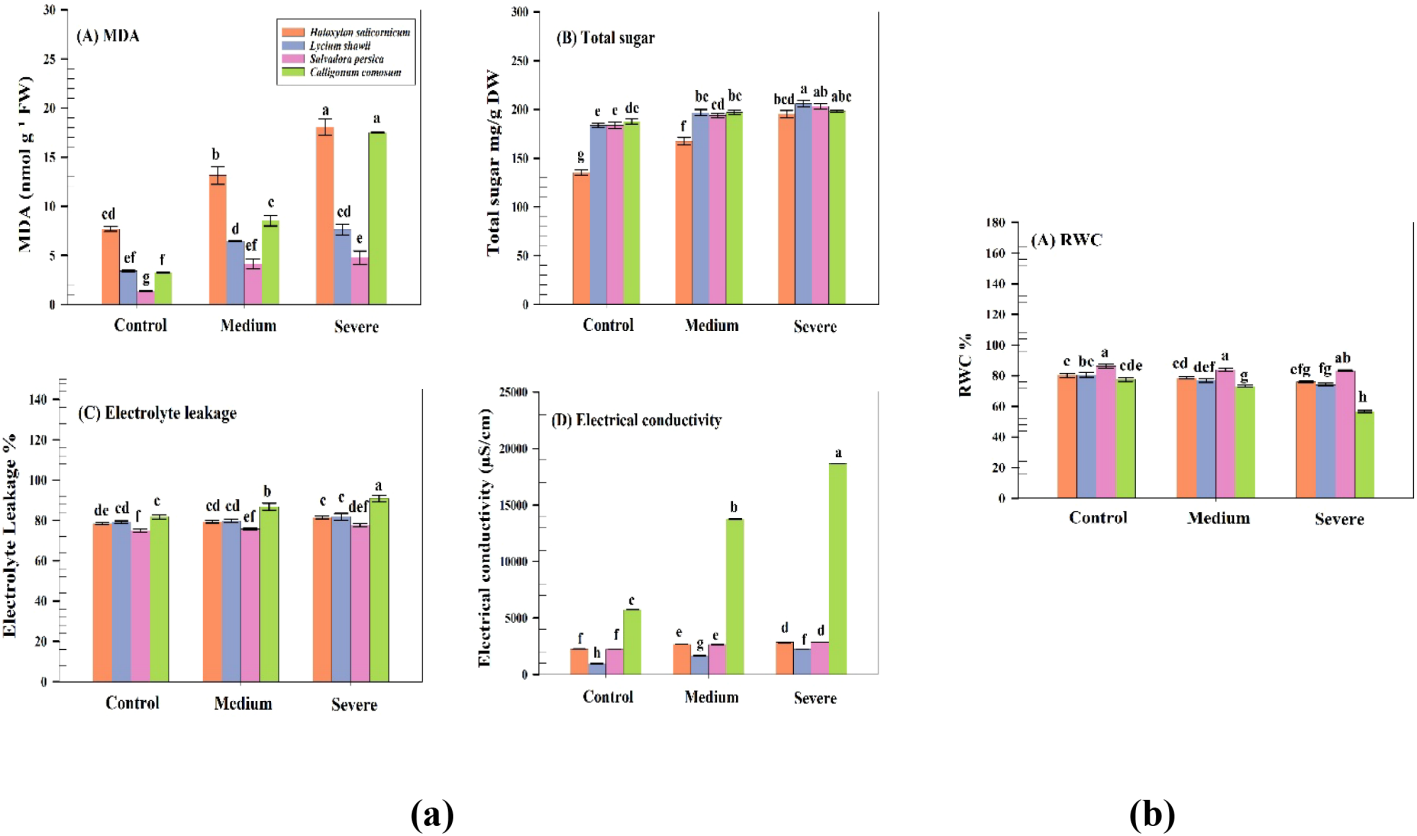
Effects of salinity on physiological and biochemical traits of four native plant species under control, moderate, and severe salinity conditions. (a) Oxidative and membrane damage–related parameters: **(A)** malondialdehyde (MDA), **(B)** total soluble sugars, **(C)** electrolyte leakage, and **(D)** leaf electrical conductivity. (b) Plant water status: **(A)** relative water content (RWC). Bars represent means ± SD (n = 5). Different lowercase letters indicate significant differences among treatments according to Tukey’s HSD test (P ≤ 0.05). Sample sizes are provided in each figure caption.

RWC differed significantly among species (P< 0.05), whereas within *S. persica* no difference was detected between control and severe salinity. *C. comosum* exhibited the largest decline (77.5 to 56.6%). *L. shawii* (80.5 to 74.0%) and *H. salicornicum* (80.2 to 75.9%) showed moderate reductions. In contrast, *S. persica* maintained relatively stable RWC (86.3 to 83.3%), with no significant difference between control and severe salinity (P > 0.05; **Figure 3bA**). A strong negative correlation was observed between RWC and electrolyte leakage (r = −0.92).

### 3.7 Effects of salinity on micro- and macronutrient content

Na^+^ accumulation varied significantly among species across all organs (**Table 3**). Under severe salinity, *H. salicornicum* showed the highest shoot Na^+^ concentration (37,333.2 mg kg^-^¹), whereas *S. persica* and *L. shawii* exhibited lower shoot Na^+^ levels. Root K^+^ declined significantly in *L. shawii* (from 4,655.6 to 3,437.6 mg kg^-^¹), whereas *C. comosum* and *H. salicornicum* maintained relatively high shoot K^+^ concentrations even under severe salinity. Phosphorus responses varied by species and organ. *C. comosum* roots showed a significant increase under moderate salinity (2,598.5 to 4,562.5 mg kg^-^¹), whereas *H. salicornicum* shoots exhibited a significant decrease under severe salinity. Micronutrient patterns also differed among species. *L. shawii* accumulated notably high Zn in shoots under severe salinity (155.2 mg kg^-^¹), while *H. salicornicum* maintained consistently high Fe and Mn concentrations in roots.

### 3.8 Effects of salinity on total soluble sugars, lipid peroxidation, and ionic homeostasis

Total soluble sugars differed significantly among species–salinity combinations (P ≤ 0.05). Under severe salinity, *L. shawii* showed the highest sugar content (206 mg g^-^¹ DW), followed by *S. persica* (203 mg g^-^¹), *C. comosum* (198 mg g^-^¹), and *H. salicornicum* (195 mg g^-^¹). Lipid peroxidation (MDA) varied significantly among species–salinity combinations (P ≤ 0.001). *C. comosum* exhibited the highest MDA levels (17.5 nmol g^-^¹ FW), whereas *S. persica* maintained the lowest (4.8 nmol g^-^¹ FW).

Electrolyte leakage (EL) differed significantly among species–salinity combinations, with the highest values recorded in *C. comosum* (91%). *L. shawii* (82%) and *H. salicornicum* (81%) showed intermediate values, whereas *S. persica* exhibited the most stable EL. Electrical conductivity (EC_leaf) varied significantly among species–salinity combinations, particularly in *C. comosum* (18,671 µS cm^-^¹; **Figure 3a D**). MDA was negatively correlated with plant height (Pearson r = −0.73) and RWC (r = −0.92), as confirmed by correlation analysis.

### 3.9 Effects of salinity on photosynthetic pigments

**Figure 4:**
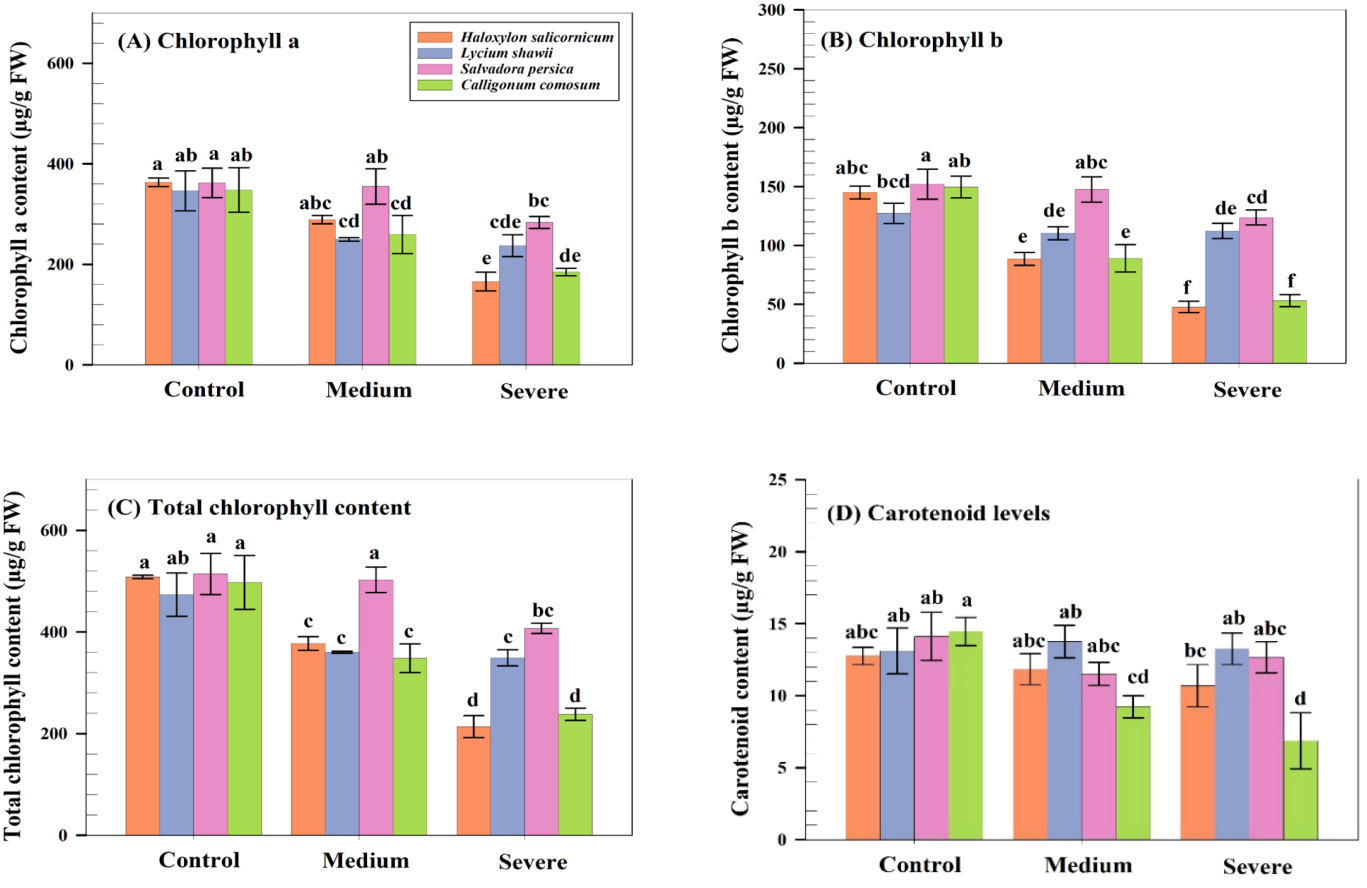
Photosynthetic pigment contents of four native shrubs under different salinity levels. **(A)** Chlorophyll (a) **(B)** Chlorophyll (b) **(C)** Total chlorophyll (µg g^-^¹ FW). **(D)** Carotenoids (µg g^-^¹ FW). Data are means ± SD (n = 5).

Chlorophyll a, chlorophyll b, total chlorophyll, and carotenoids differed significantly among species (p ≤ 0.05). *C. comosum* exhibited the lowest total chlorophyll under severe salinity (237.3 µg g^-^¹ FW). *H. salicornicum* showed a marked reduction in chlorophyll a (165.7 µg g^-^¹ FW) and carotenoids (10 µg g^-^¹ FW). In contrast, *L. shawii* maintained relatively stable pigment levels, with only moderate declines in chlorophyll a and no significant change in carotenoids (13.1 to 13.3 µg g^-^¹ FW). *S. persica* displayed intermediate reductions in total chlorophyll (407.2 µg g^-^¹ FW; **Figure 4**).

### 3.10 Effects of salinity on photosynthetic parameter

**Figure 5:**
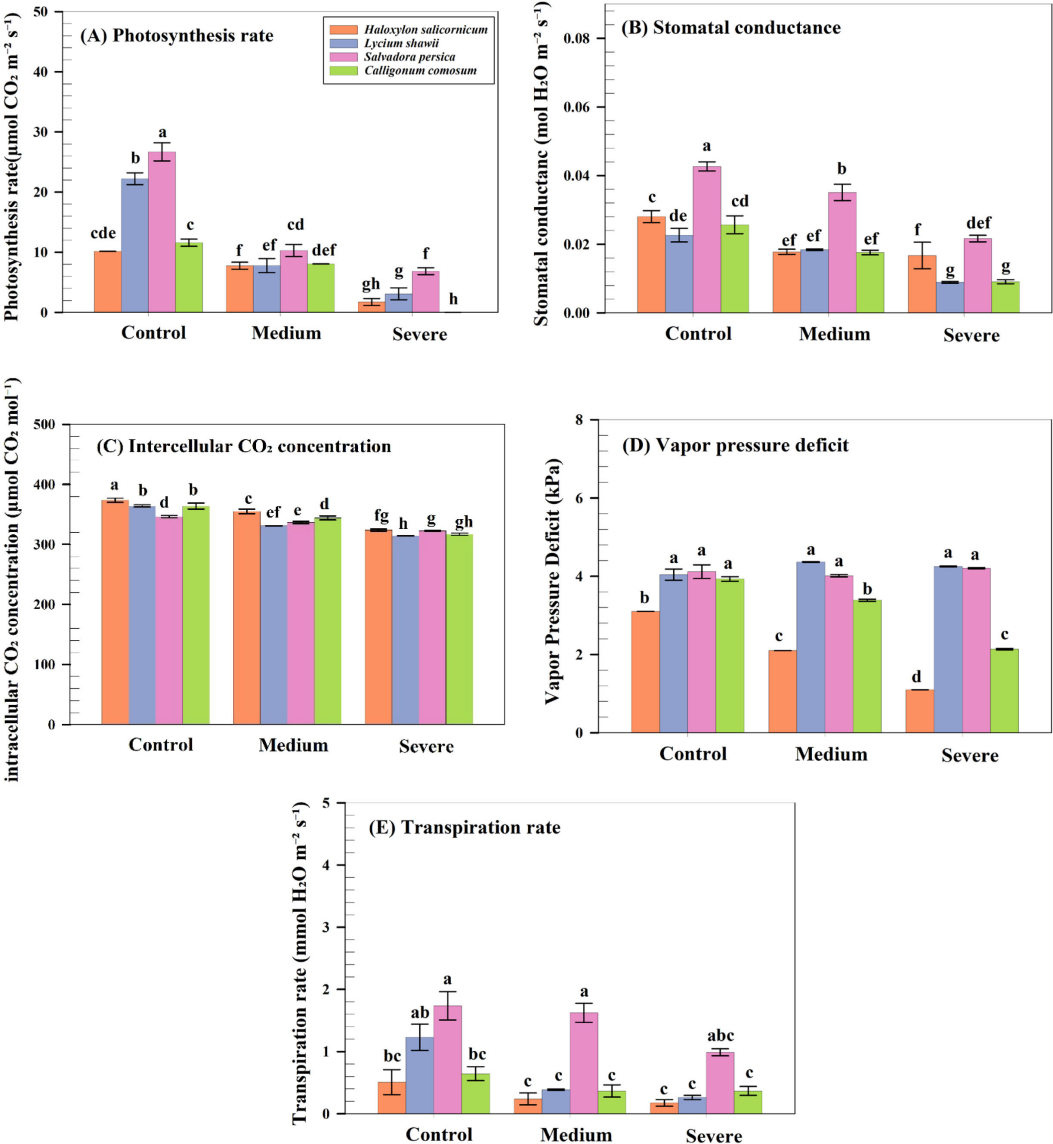
Gas-exchange responses of four native shrubs under salinity. **(A)** Net photosynthetic rate [**(A)**, µmol CO_2_ m^-^² s^-^¹]. **(B)** Stomatal conductance (gs, mol m^-^² s^-^¹). **(C)** Intercellular CO_2_ concentration (Ci, µmol mol^-^¹). **(D)** Leaf vapor pressure deficit (VPDleaf, kPa). **(E)** Transpiration rate (E, mmol m^-^² s^-^¹). Data are means ± SD (n = 3).

All gas exchange parameters varied significantly among species (P< 0.05). Under severe salinity, S. persica exhibited the highest photosynthetic rate (7.18 µmol CO_2_ m^-^² s^-^¹), followed by *L. shawii* (4.08), while *H. salicornicum* (2.06) and *C. comosum* showed lower rates. Stomatal conductance was highest in *S. persica* (0.043 mol m^-^² s^-^¹). Intercellular CO_2_ concentration was significantly reduced in *H. salicornicum* and *L. shawii*. VPDleaf was higher in *S. persica* and *L. shawii* (>4.2 kPa), whereas *H. salicornicum* maintained lower VPDleaf (1 kPa). Transpiration rate followed a similar pattern, being highest in *S. persica* (2.20 mmol m^-^² s^-^¹) and lowest in *H. salicornicum* (0.33 mmol m^-^² s^-^¹; **Figure 5**).

### 3.11 Effects of salinity on DPPH and antioxidant activity

**Figure 6:**
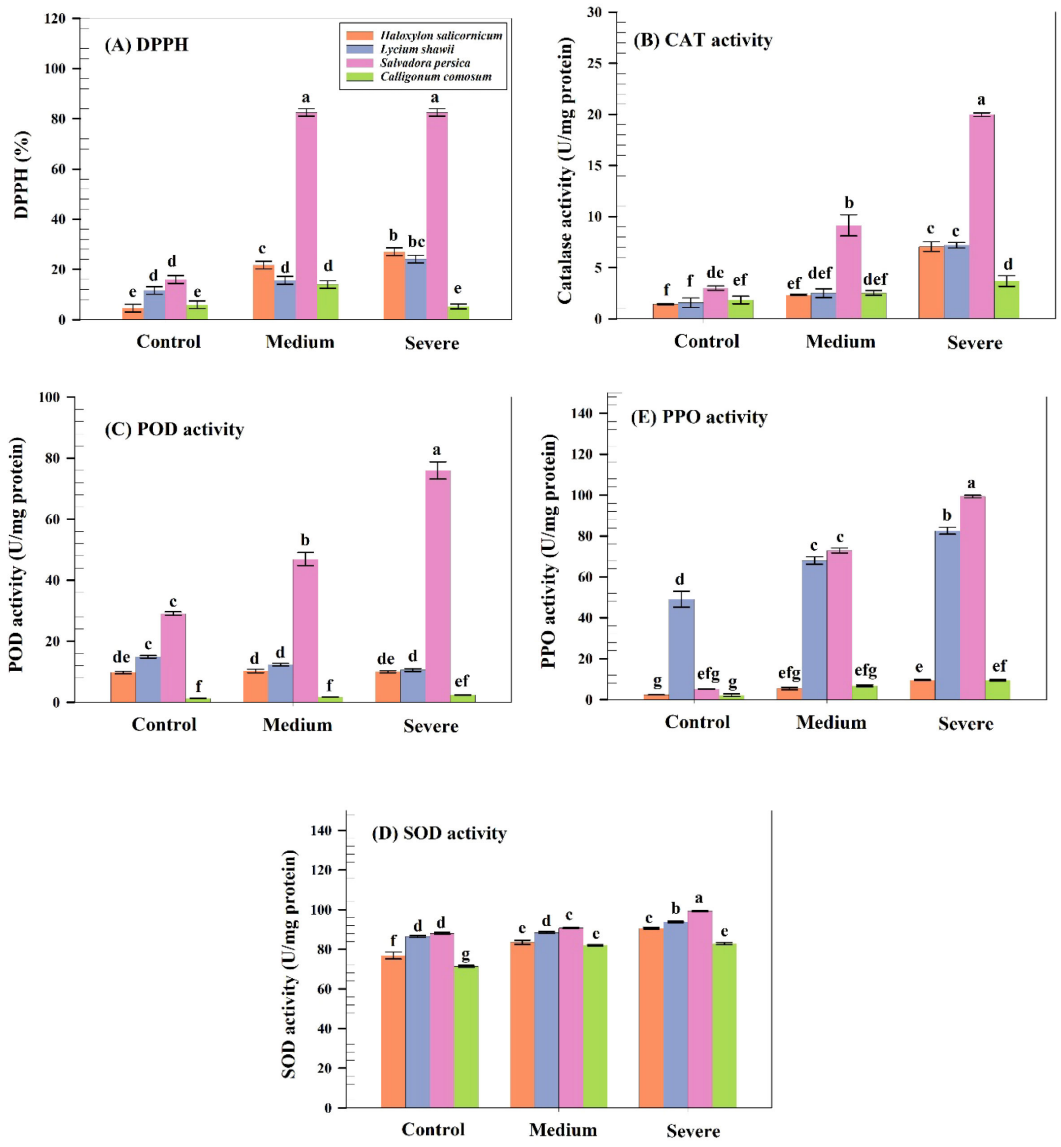
Antioxidant responses of the studied species under control, moderate, and severe salinity. **(A)** DPPH radical scavenging activity (%). **(B)** Catalase (CAT) activity. **(C)** Peroxidase (POD) activity. **(D)** Superoxide dismutase (SOD) activity. **(E)** Polyphenol oxidase (PPO) activity. Values are means ± SD (n = 6).

Non-enzymatic antioxidant activity differed significantly among species (p ≤ 0.05). *S. persica* exhibited the highest DPPH scavenging activity (83.9%), followed by *H. salicornicum* (28.4%) and *L. shawii* (25.4%), whereas *C. comosum* showed minimal activity (6.3%). *S. persica* displayed the highest CAT (20.15 U mg^-^¹ protein), POD (84.3), SOD (99.5), and PPO (100) activities under severe salinity. *L. shawii* exhibited intermediate enzyme activities, whereas *C. comosum* consistently showed the lowest CAT, POD, and PPO activities (**Figure 6**).

### 3.12 Effects of salinity on proline, oxidative markers, total phenolic content, and protein levels

**Figure 7:**
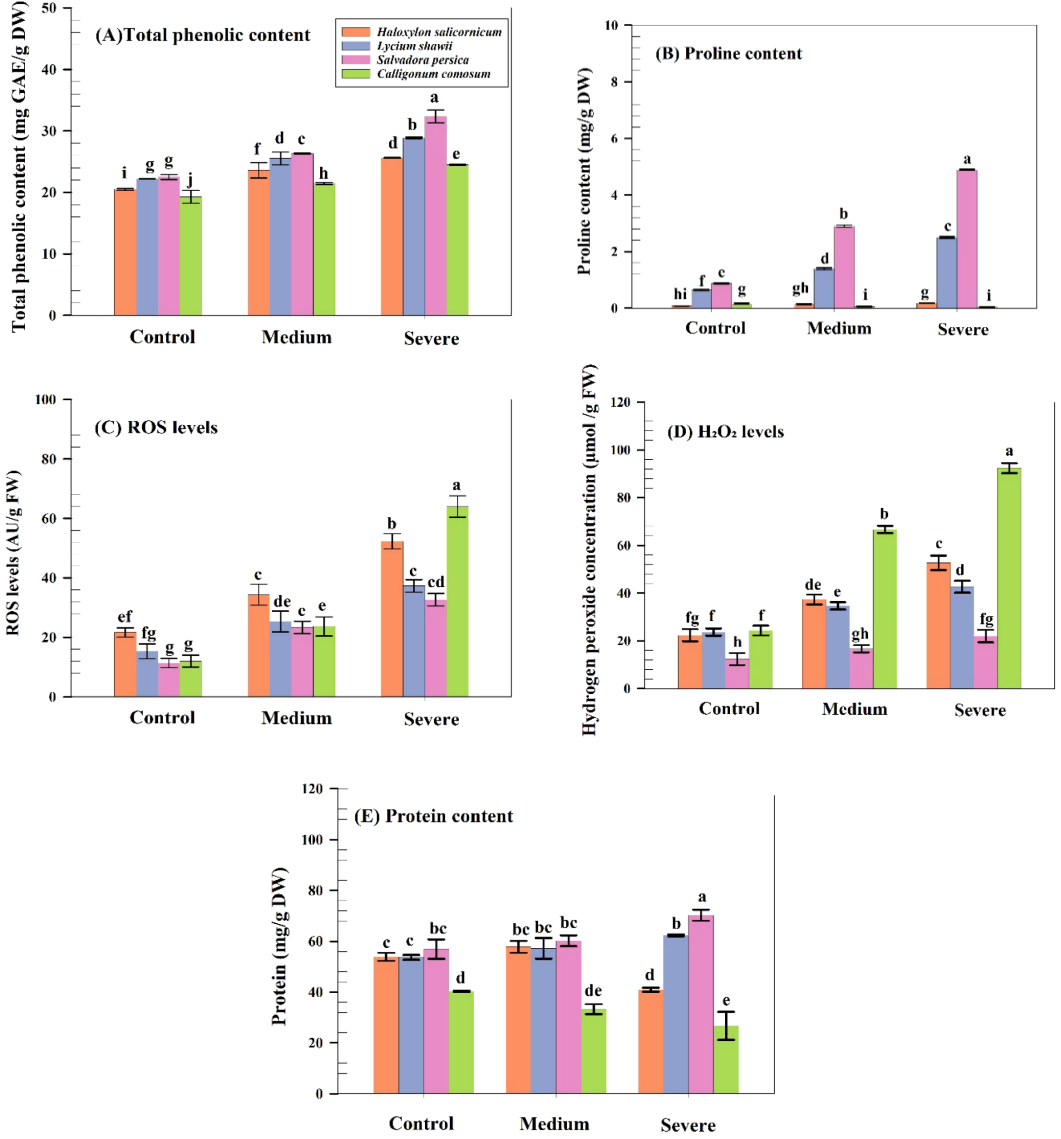
Biochemical and oxidative stress indicators in four native shrubs under salinity. **(A)** Total phenolic content (mg GAE g^-^¹ DW). **(B)** Proline content (mg g^-^¹ DW). **(C)** Reactive oxygen species (ROS, AU/g FW). **(D)** Hydrogen peroxide (H_2_O_2_, µmol g^-^¹ FW). **(E)** Total soluble protein (mg g^-^¹ DW). Values are means ± SD (n = 6).

Total phenolic content varied significantly among species (p ≤ 0.05). *S. persica* showed the highest phenolic levels (32.3 mg GAE g^-^¹ DW), followed by *L. shawii* and *H. salicornicum*, while *C. comosum* had the lowest (24.5 mg GAE g^-^¹ DW). Proline increased significantly in *S. persica* (4.9 mg g^-^¹ DW) and *L. shawii* (2.5 mg g^-^¹ DW) but declined sharply in *C. comosum* (0.05 mg g^-^¹ DW). Total protein content increased under severe salinity in *S. persica* (72.6 mg g^-^¹ DW) and *L. shawii* (59.9 mg g^-^¹ DW) but decreased in *C. comosum* (25.4 mg g^-^¹ DW). ROS and H_2_O_2_ levels differed significantly among species–salinity combinations (P ≤ 0.05). *C. comosum* exhibited the highest ROS (64 AU/g FW) and H_2_O_2_ (92 µmol g^-^¹ FW), whereas *S. persica* and *L. shawii* showed lower oxidative stress levels (**Figure 7**).

### 3.13 Correlation analysis and principal component analysis

**Figure 8:**
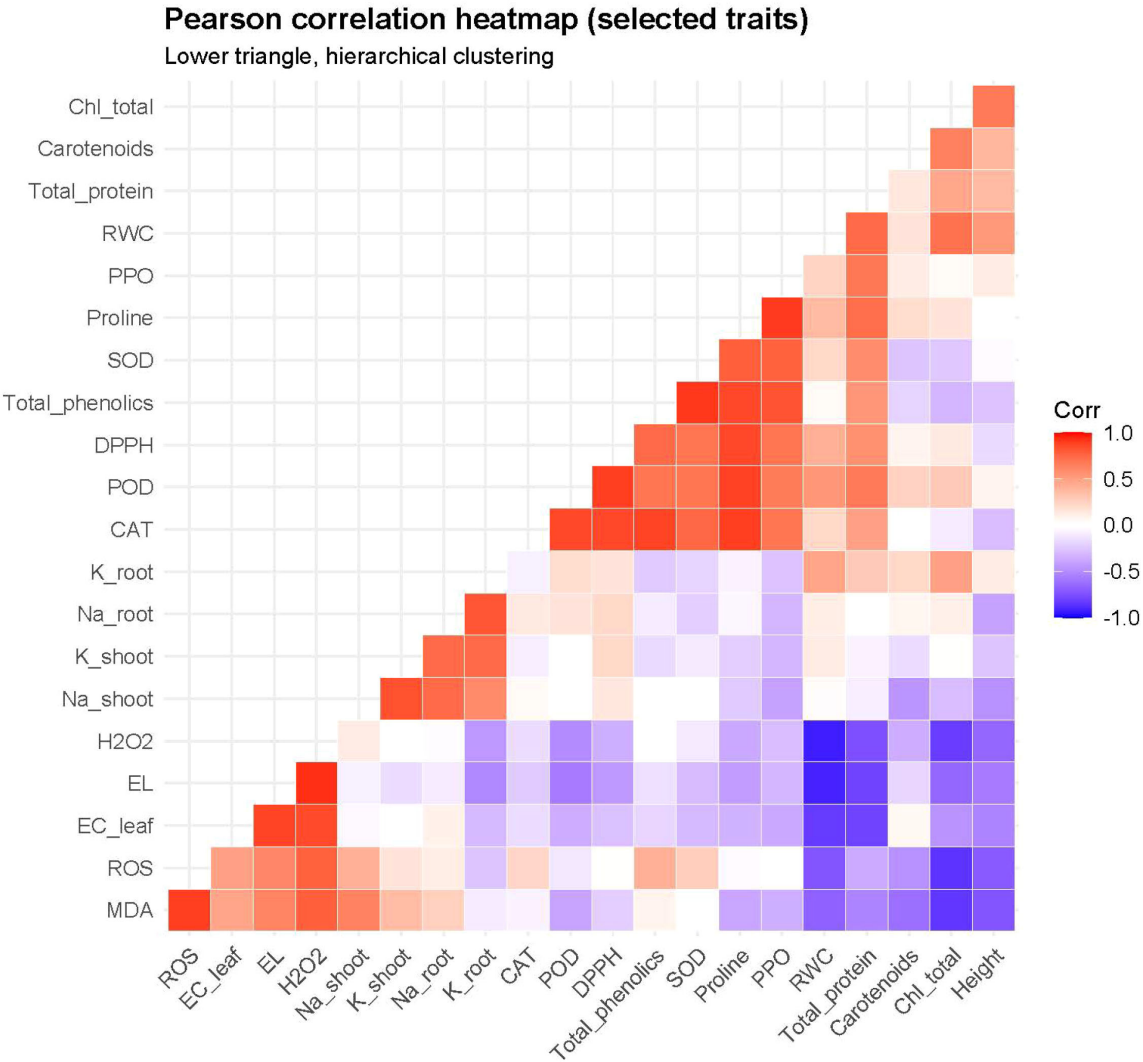
Pearson correlation heatmap illustrating relationships among selected physiological, biochemical, and ionic traits under salinity. The heatmap represents the lower triangular correlation matrix with hierarchical clustering applied to variables using Ward’s method and Euclidean distance. Warmer colours (red) denote positive correlations, whereas cooler colours (blue) indicate negative correlations, with colour intensity reflecting the magnitude of correlation coefficients.

Pearson correlation heatmap revealed clear associations among physiological and biochemical traits under salinity stress (**Figure 8**). Oxidative stress indicators (ROS, MDA, H_2_O_2_, electrolyte leakage, and EC_leaf) were strongly positively correlated with each other and negatively correlated with plant performance traits including plant height and relative water content. Notably, RWC showed a strong negative correlation with electrolyte leakage (r = −0.92), and plant height was negatively correlated with MDA (r = −0.73), confirming the impact of membrane damage and oxidative stress on growth reduction. Conversely, osmoprotectants and defense metabolites (proline, total phenolics, antioxidant enzymes, and protein content) showed strong positive relationships, with proline highly correlated with total phenolics (r = 0.86) and total protein (r = 0.72), indicating coordinated biochemical protection mechanisms. Ion homeostasis traits formed a distinct cluster: Na^+^ accumulation was associated with oxidative stress variables, whereas K^+^ retention was positively related to photosynthetic pigments and growth traits, suggesting its role in maintaining physiological stability under salinity. This pattern corresponded closely with PCA clustering, confirming that oxidative damage variables drive species separation under salinity stress.

**Figure 9:**
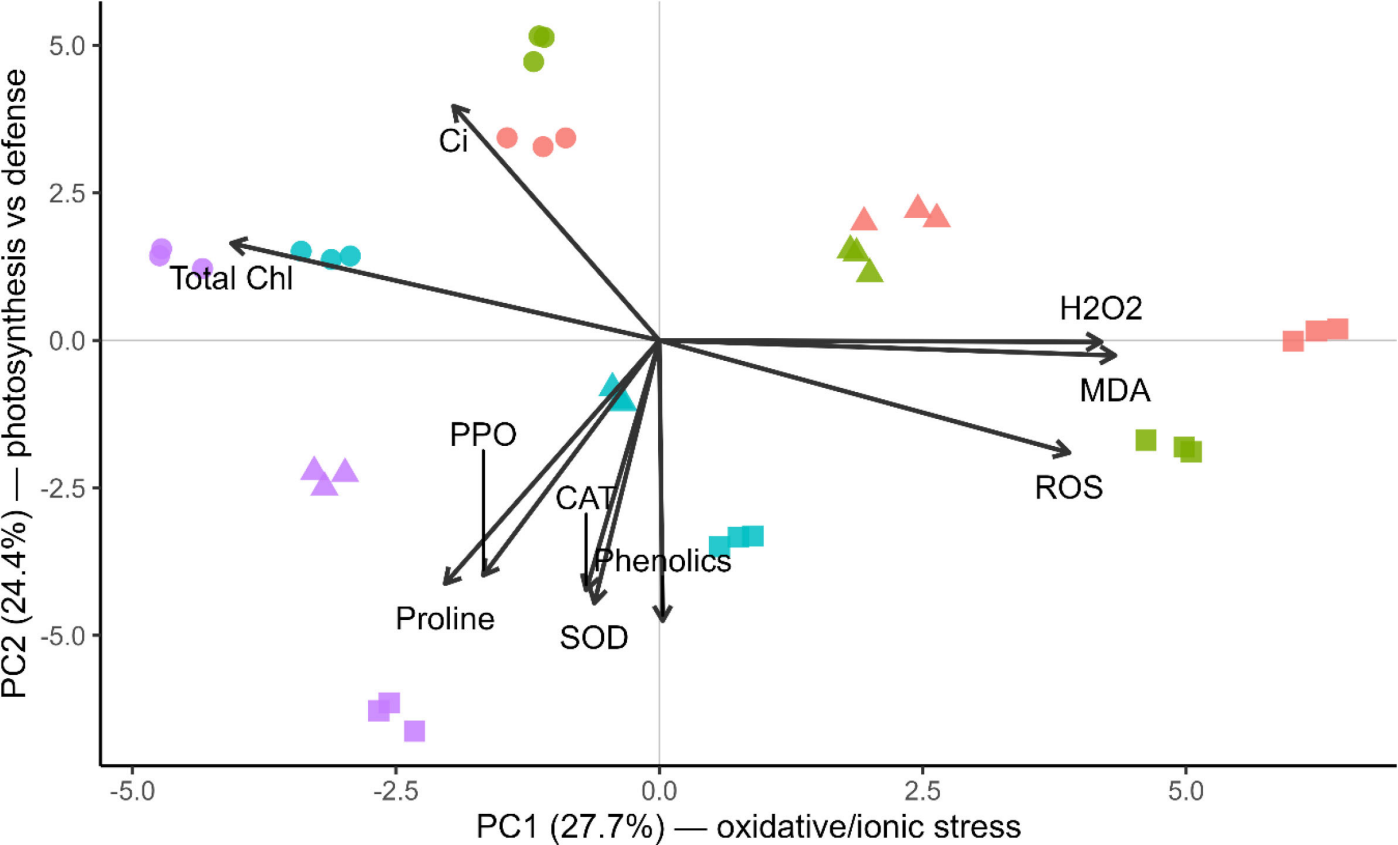
Principal component analysis (PCA) biplot showing relationships among physiological and biochemical traits under salinity stress. Arrows indicate variable loadings. PC1 represents an oxidative–ionic stress gradient, while PC2 represents biochemical defence activity. Coloured points represent plant species: *Calligonum comosum*, *Haloxylon salicornicum*, *Lycium shawii*, and *Salvadora persica*. Species separation reflects distinct salt-tolerance strategies, ranging from oxidative damage-dominated response to maintenance of photosynthetic stability and antioxidant protection. Colored symbols indicate species identity: *C. comosum* (purple triangles), *H. salicornicum* (green squares), *L. shawii* (cyan circles), and *S. persica* (red triangles).

Principal component analysis (PCA) biplot (**Figure 9**) further integrated these relationships. The first two principal components explained 27.7% (PC1) and 24.4% (PC2) of total variance. PC1 represented an oxidative–ionic stress gradient, positively associated with ROS, MDA, and H_2_O_2_ and negatively associated with chlorophyll and internal CO_2_ concentration (Ci). PC2 represented biochemical defense activation, associated with proline accumulation, antioxidant enzymes (SOD, CAT, PPO), and phenolic compounds.

Species separation reflected distinct tolerance strategies. In the biplot, species were distinguished by color: *C. comosum* (purple), *H. salicornicum* (green), *L. shawii* (cyan), and *S. persica* (red). *C. comosum* clustered within the oxidative stress region, indicating susceptibility to salinity. *H. salicornicum* occupied an intermediate ionic-adjustment region. *L. shawii* was positioned along the biochemical defense axis, showing osmotic and antioxidant adaptation. *S. persica* grouped with photosynthetic integrity traits, maintaining chlorophyll and lower oxidative damage.

Together, the heatmap and PCA demonstrate that salinity tolerance among species is governed by three coordinated adaptive strategies: oxidative stress avoidance, ionic regulation, and biochemical defense activation.

## 4 Discussion

Exposure to salinity resulted in species-specific responses that reflected contrasting tolerance strategies among the studied native shrubs. All species survived under control and moderate salinity conditions; however, under severe salinity, *Calligonum comosum* exhibited a reduced survival rate (83.3%), indicating comparatively lower tolerance (Latef et al., 2023). This reduced performance aligns with previous findings suggesting that *C. comosum* is primarily adapted to arid, sandy environments rather than highly saline soils, relying more on structural and mechanical adaptations than biochemical salt tolerance mechanisms (Soliman et al., 2018; Latef et al., 2023). In contrast, *Salvadora persica* and *Lycium shawii* maintained full survival with minimal salt injury index (SI: 1–1.5), demonstrating superior tolerance. Survival alone did not reflect physiological tolerance differences among species, as substantial sub-lethal damage occurred in less tolerant species and that physiological injury indices provide deeper insights into hidden salinity effects (**Table 4**).

Plant height differed significantly among species–salinity combinations (P< 0.05), with the greatest reduction observed in *C. comosum* under severe salinity (50.2%), likely due to combined osmotic stress and ion toxicity that inhibited cell expansion and shoot elongation. In contrast, *S. persica* and *L. shawii* exhibited better growth maintenance, possibly related to hormonal regulation (e.g., ABA and ethylene signaling) (Müller, 2021; Lim et al., 2022; Nazir et al., 2024). Leaf area and stem development were also differentially affected. *S. persica* showed the least reduction in leaf area, suggesting efficient osmotic protection and ion exclusion mechanisms, while *C. comosum* experienced marked stem reduction, consistent with its weaker tolerance (Mann et al., 2023). Biomass allocation patterns further supported species-specific adaptation strategies. *S. persica* and *L. shawii* increased root-to-shoot ratios under high salinity, indicating a strategic shift toward enhanced water and nutrient acquisition. This pattern is consistent with salt-tolerant species such as *Saussurea salsa*, which similarly increase root investment under salinity stress as an adaptive survival mechanism (Arif et al., 2020; Monson et al., 2022; Li et al., 2021; Bao et al., 2025).

Relative water content (RWC) declined in all species with increasing salinity, but to varying extents. *S. persica* maintained the most stable RWC (83.3%), likely due to deep rooting, effective osmotic adjustment, and strong biochemical defenses. *H. salicornicum* also maintained relatively high RWC, attributed to its succulent tissues that dilute internal salt concentrations and reduce transpiration. In contrast, *C. comosum* exhibited the sharpest RWC decline (56.6%), reflecting limited osmotic adjustment and ion exclusion capacity. These findings align with previous studies on *Atriplex halimus*, where populations with stronger osmotic regulation showed improved water-use efficiency under salinity (Martinez et al., 2003).

Salinity altered nutrient uptake and ion compartmentalization in all species. Sodium (Na^+^) accumulation increased across species but was particularly pronounced in *H. salicornicum* and *S. persica*, likely due to efficient vacuolar sequestration mechanisms that help maintain cytosolic ionic balance. *L. shawii* exhibited a decline in root K^+^ under severe salinity, reflecting Na^+^–K^+^ competition at uptake sites. However, *S. persica* and *C. comosum* maintained relatively high shoot K^+^ levels, suggesting selective K^+^ transport and Na^+^ compartmentalization (Malakar and Chattopadhyay, 2021; Sun et al., 2024). These findings are consistent with salt-tolerant crops such as cotton, where genotypes exhibiting better Na^+^ sequestration and higher K^+^ retention demonstrate enhanced salinity tolerance through improved ionic homeostasis (Sun et al., 2021).

Chlorophyll and carotenoid content varied significantly among species, particularly in *C. comosum*, likely due to activation of chlorophyll-degrading enzymes and downregulation of pigment biosynthesis genes (Wang et al., 2024). In contrast, *L. shawii* maintained pigment stability, reflecting stronger antioxidant protection (Li et al., 2022). Gas exchange parameters declined in all species. *C. comosum* showed almost complete inhibition of photosynthesis, whereas *S. persica* maintained the highest photosynthetic rate under severe salinity, likely due to efficient stomatal regulation and osmotic adjustment. *H. salicornicum* adopted a conservative water-use strategy through ABA-mediated stomatal closure, reducing water loss but limiting carbon assimilation. These patterns support previous findings in sorghum and other salt-tolerant species that rely on coordinated stomatal and osmotic regulation to maintain physiological function under salinity (Sakoda et al., 2021; Dourado et al., 2022).

Proline accumulation increased significantly in *S. persica* and *L. shawii*, indicating strong osmotic adjustment capacity, likely mediated by upregulation of P5CS activity. Proline likely contributed to membrane stabilization, ROS detoxification, and redox balance. In contrast, *C. comosum* showed a sharp decline in proline content, suggesting poor osmotic regulation (Maghraby and Alzalaty, 2025). Similar patterns of proline accumulation have been reported in turfgrass, where enhanced proline levels mitigate dehydration and oxidative damage (Jiang et al., 2023). Total soluble sugars increased in all species under salinity, with *L. shawii* exhibiting the highest accumulation. These sugars likely contributed to osmotic balance and acted as signaling molecules regulating stress-responsive pathways. However, in *C. comosum*, sugar accumulation was insufficient to counteract pigment degradation and metabolic disruption (Afzal et al., 2021; Nawaz et al., 2022).

Salinity led to increased ROS and H_2_O_2_ accumulation in all species, with *C. comosum* showing the highest levels due to weaker antioxidant defenses. *S. persica* exhibited the strongest enzymatic antioxidant response (high CAT and POD activity), effectively mitigating oxidative damage. This pattern aligns with findings in salt-tolerant maize genotypes that exhibit stronger ROS-scavenging systems (Qiao et al., 2021; Rohman et al., 2024).

Lipid peroxidation (MDA) was highest in *C. comosum*, reflecting membrane instability, while *S. persica* maintained the lowest MDA levels, indicating superior oxidative stress tolerance. Total phenolic content increased across all species, but was highest in *S. persica*, further supporting its strong non-enzymatic antioxidant capacity. These results are consistent with a previous study in *Catharanthus roseus*, which maintains low MDA under stress through enhanced antioxidant defense (Ali et al., 2021). Protein content increased in *L. shawii* and *S. persica* under severe salinity, likely due to synthesis of stress-responsive proteins such as LEA proteins, which protect cellular structures during dehydration (Abdul Aziz et al., 2021; Hsiao, 2024).

Correlation analysis confirmed that ionic toxicity and osmotic stress were major constraints under high salinity, as indicated by strong negative relationships between RWC and electrolyte leakage. Furthermore, multivariate analysis provided integrative evidence for the mechanistic interpretation of salinity tolerance. The correlation heatmap revealed two clearly opposing functional networks: a stress-damage cluster (ROS, MDA, H_2_O_2_, electrolyte leakage, and EC_leaf) and a protection cluster (proline, phenolics, antioxidant enzymes, and protein content). The strong negative associations between oxidative markers and growth traits confirmed that membrane injury and oxidative imbalance were the primary drivers of growth inhibition under salinity rather than osmotic limitation alone.

The PCA supported this interpretation by separating species along two principal functional axes. PC1 represented the oxidative–ionic stress gradient, distinguishing the highly damaged *C. comosum* from the more stable species, whereas PC2 represented biochemical defense activation associated with osmotic adjustment and antioxidant protection. *S. persica* clustered with photosynthetic stability traits, *L. shawii* with biochemical protection mechanisms, and *H. salicornicum* occupied an intermediate ionic-regulation position. This multivariate separation confirms that salinity tolerance in native desert shrubs is governed by coordinated but species-specific physiological strategies rather than a single tolerance mechanism. Positive correlations among proline, phenolics, and protein content highlight the coordinated role of osmotic adjustment and antioxidant defense in salt-tolerant species. *C. comosum* in its native habitat prefers dune slopes and semi-stabilized sands, where transient droughts occur but salinity remains moderate. Similar trends were reported in *Calligonum caput-medusae* under water deficit (Wang et al., 2024), highlighting the genus’s reliance on opportunistic growth rather than constitutive salt tolerance. These findings are consistent with earlier drought-focused assessments of UAE desert species, where *L. shawii* and *S. persica* showed superior tolerance through coordinated physiological, biochemical, and structural adaptations (Abo Amin et al., 2025).

The findings highlight *S. persica* and *L. shawii* as strong candidates for saline land rehabilitation and afforestation programs in arid regions. These species offer ecological benefits, including erosion control, biodiversity enhancement, and habitat provision, as well as agricultural benefits such as forage production and potential use in salt-tolerant crop breeding. However, large-scale implementation requires efficient propagation methods such as tissue culture and cost-effective restoration strategies.

Unlike previous studies that examined these species individually or under controlled greenhouse conditions, the present work demonstrates that salinity tolerance in UAE native shrubs is not governed by a single mechanism but by three coordinated strategies (ionic regulation, biochemical defense activation, and oxidative stress avoidance), and that species occupy distinct functional tolerance niches under real desert environmental conditions.

This study provides a comprehensive comparative assessment of physiological, biochemical, and ionic responses of four key UAE native shrubs as an outdoor pot trial under ambient desert conditions salinity treatments. The results advance understanding of species-specific salinity tolerance mechanisms and providing an ecological-physiological framework for selecting native species in desert salinity rehabilitation and biosaline agriculture programs.

## 5 Conclusion

Among the studied species, *S. persica* and *L. shawii* had the highest tolerance to salinity stress, maintaining better water status, photosynthetic performance, and antioxidant protection. In contrast, *C. comosum* showed pronounced physiological damage, indicating limited tolerance.

This study provides an integrated comparison of salinity adaptation mechanisms in these native species and highlights *S. persica* and *L. shawii* as promising candidates for saline land rehabilitation, biosaline agriculture, and ecological restoration in arid regions. These traits may provide useful targets for future breeding and molecular studies aimed at improving crop salinity tolerance. Future work should validate the underlying molecular mechanisms and support large-scale propagation to facilitate practical deployment.

## Data Availability

The original contributions presented in the study are included in the article/supplementary material. Further inquiries can be directed to the corresponding author.
